# Eight Voices in Darkness: using art to involve the public in research dissemination

**DOI:** 10.1186/s40900-026-00951-z

**Published:** 2026-08-06

**Authors:** Claire Nollett, Richard Bowers, Nicki Cockburn, Rhys Denton, Keziah Latham

**Affiliations:** 1 Centre for Vision Services Research, Cardiff University, Maindy Road, Cardiff, CF24 4HU UK; 2 http://www.richardbowers.co.uk; 3 Cardiff, UK; 4 Centre for Trials Research, Cardiff University, 7th Floor Neuadd Meirionnydd, University Hospital of Wales, Heath Park, Cardiff, CF14 4YS UK; 5 Research Centre for Better Living, Anglia Ruskin University, Cambridge, CB1 1PT UK

**Keywords:** Patient involvement, Public involvement, Public engagement, Vision impairment, Sight loss, Art, Mental health, Dissemination

## Abstract

**Background:**

Sight loss will affect an estimated 2.7 million people in the UK by 2030 and is associated with substantial reductions in quality of life, mobility, independence, and poorer mental health. Our own research has demonstrated that depressive symptoms are highly prevalent among blind and partially sighted people yet often remain unrecognised and untreated. To effectively disseminate our research to healthcare professionals, we involved members of the public with lived experience of sight loss in an art installation. The aim of this project is to evaluate the feasibility and value of using such an arts-based approach as a method for involving blind and partially sighted individuals in dissemination, and here we reflect on what all stakeholders learnt from the process.

**Main body:**

We recruited eight blind and partially sighted storytellers to share themes from our research using narratives of their own lived experience, focusing on the emotional impact of sight loss and professional interactions. Audio-recorded stories and accompanying portraits were developed into Eight Voices in Darkness, a sound installation designed to reflect both the literal and metaphorical darkness associated with vision impairment and depression. The installation was exhibited publicly over three days alongside workshops for eyecare professionals and mental health practitioners. Visitor and participant feedback (n = 92) was analysed using Pendleton’s reflective framework to explore what went well, what did not, and what was learned.

Visitors consistently reported an enhanced understanding of the mental health consequences of sight loss, valuing the authenticity, emotional resonance, and individuality conveyed through the artistic format. Many indicated intentions to adopt more empathetic, patient centred practices, improve accessibility, and initiate conversations about mental health. Storytellers highlighted the value of being heard and connecting with others’ experiences. Researchers and the artist reflected on the strengths of partnership with the charity, the power of the immersive format, and challenges relating to managing expectations. The process generated important insights into involving the public in dissemination through art, including ethical editing, audience preparation, and sustaining involvement.

**Conclusion:**

Involving people with lived experience in arts-based dissemination offers a powerful and engaging method for disseminating research findings to professionals to improve healthcare. Future work should refine practical elements and enhance coproduction.

## Abstract (plain language summary)

Many people in the UK live with sight loss, which can make every aspect of everyday life harder, including tasks like reading and mobility. Our research showed that sight loss can also impact mental health, and that many people with sight loss do not get the emotional support they need, partly because some eyecare professionals do not realise how common mental health problems are among this group. Our aim was to involve patients and the public, specifically blind and partially sighted people, in sharing our research effectively so that vision-related professionals and the public could learn how sight loss affects mental health. We created an art project called *Eight Voices in Darkness*. Eight individuals talked about the key topics noted in our previous body of research on sight loss and mental health: how their sight loss affects their feelings, challenges faced, and support required. Their stories were recorded, along with a portrait photo of each person. An artist turned these stories into an art installation. In a quiet, dark theatre, visitors could sit and listen to short parts of each person’s story. Over three days, almost 200 people visited. Many said the experience helped them understand sight loss in a new and powerful way and they would try to be more kind, patient, and willing to talk about mental health in the future. The storytellers also said it felt good to be listened to. This project showed that art is a valuable mechanism to involve individuals with lived experience in sharing research with professionals and the public to improve healthcare. Research teams must consider practical and ethical considerations when individuals’ stories are harnessed to share research findings and allow sufficient time and resources.

## Background

By 2030, 2.7 M UK individuals will live with some form of sight loss, leading to reduced quality of life, mobility and independence and poorer mental health [1]. People with sight loss have higher prevalence of depression [2–4], anxiety [3], and Post Traumatic Stress Disorder [5] than people without sight loss. Our own research demonstrates that the prevalence of depressive symptoms in blind and partially sighted people in the UK is 43% (compared to 16% of the general population and 35% of individuals with a disability in Great Britain [6]); and of those with depression, 75% were not in receipt of any treatment [2]. This is partly due to lack of mental health consideration by professionals: for example, only a third of low vision practitioners (optometrists and dispensing opticians who had completed a further Professional Certificate in Low Vision) in Wales actively tried to identify depression in their patients [7].

There is therefore a need to disseminate our research more widely than through academic publications, to educate professionals in the sight loss sector (e.g. ophthalmologists, Low Vision Rehabilitation Workers, Eye Clinic Liaison Officers, charity staff) about the link between vision impairment and poor mental health. Public dissemination would also be beneficial, as the UK public have a poor understanding of sight loss in general [8].

Within this project, based on the National Institute for Health and Care Research (NIHR) School for Public Health Research (SPHR) Public Mental Health (PMH) Programme definitions [9] we define public involvement as actively involving individuals with lived experience of vision impairment and mental health issues in the dissemination of research.

Despite growing expectations for public involvement across the research cycle, involvement in dissemination is still often limited, tokenistic, or poorly evaluated. In particular, there is limited evidence on how to meaningfully involve people with lived experience in shaping how research findings are communicated to professional audiences, and what difference this makes to learning, attitudes, or practice. Involvement in dissemination moves beyond communicating findings “to” the public, towards working “with” people to shape messages, formats and priorities, potentially increasing relevance, credibility and impact.

Hearing from people with lived experience has been shown to have positive impacts on healthcare professionals including: increased awareness of patient perspectives and experiences, greater empathy, enhanced communication skills, self-reflection and personal growth [10]. Digital stories can be a powerful tool for dissemination, knowledge transfer and education in healthcare settings [11]. However, meaningful involvement requires careful attention to issues of power, representation, emotional labour and support, particularly when sharing personal stories about health and wellbeing.

Our aim was to involve individuals with lived experience in disseminating our research by combining digital stories with an immersive arts-based approach. This project therefore addresses an important gap by exploring how people with lived experience can be actively involved in dissemination through an arts-based, immersive format, and what is learned by contributors, researchers, and audiences through this process. The framework used for reflection is Boud’s triangular representation [12] (Experience; Reflection; Learning), with the reflective element utilising the reflection questions from Pendleton’s model [13].

## Experience

Arts-based approaches use various forms of art, including theatre, dance, photography and poetry to disseminate research findings [14, 15], with visual forms of art being popular dissemination tools e.g. [16]. However, we utilised mainly audio content to reflect the experiences of people with sight loss. A sound installation was an immersive way to share the stories of eight blind and partially sighted people, followed by workshops to reflect on learning for vision-related and mental health professionals. We also created a largely audio based digital video to be hosted online, to disseminate the work nationally and internationally.

We partnered with Sight Life, a local charity supporting people with sight loss, to identify and involve eight blind and partially sighted people in the art installation as storytellers. We were inclusive of different genders, age, eye conditions and level of sight loss and included: three men and five women; five individuals with visual impairment from birth and three who lost sight later in life; five who are blind and three who are partially sighted; three guide dog users. Author CN (a Psychological Wellbeing Practitioner and mental health researcher) held an introductory meeting to explain the project and our research findings. Individuals were given an information sheet (in large print or audio) and asked to give written or verbal consent to have their story audio recorded and shared. CN met each storyteller individually in a place of their choosing, which included the charity’s recording studio, at their home or in a park. Story elicitation was guided by open prompts based around our findings to include information about their sight loss, impact on mental health, support received (or not) and what professionals could do differently. Storytellers were free to say as much or as little as they wished, and conversations lasted up to an hour. A member of Sight Life’s photography club took a portrait photograph of each person to accompany their story in the installation (or two people supplied their own). Individuals were reimbursed for their involvement in line with NIHR recommended rates [17].

We aimed to present an hour’s worth of material in the installation and to include each storyteller, hence the stories required editing. Authors CN and KL (a research optometrist) reviewed the transcripts to select excerpts which best represented and conveyed the key research findings e.g. sight loss leading to depression or anxiety and lack of detection and support offered in eye care services [2, 7], as well as key symptoms [18] and triggers to improve future detection. The materials were securely transferred to author RB, an installation artist who has experience of working with blind and partially sighted individuals.

**Fig. 1:**
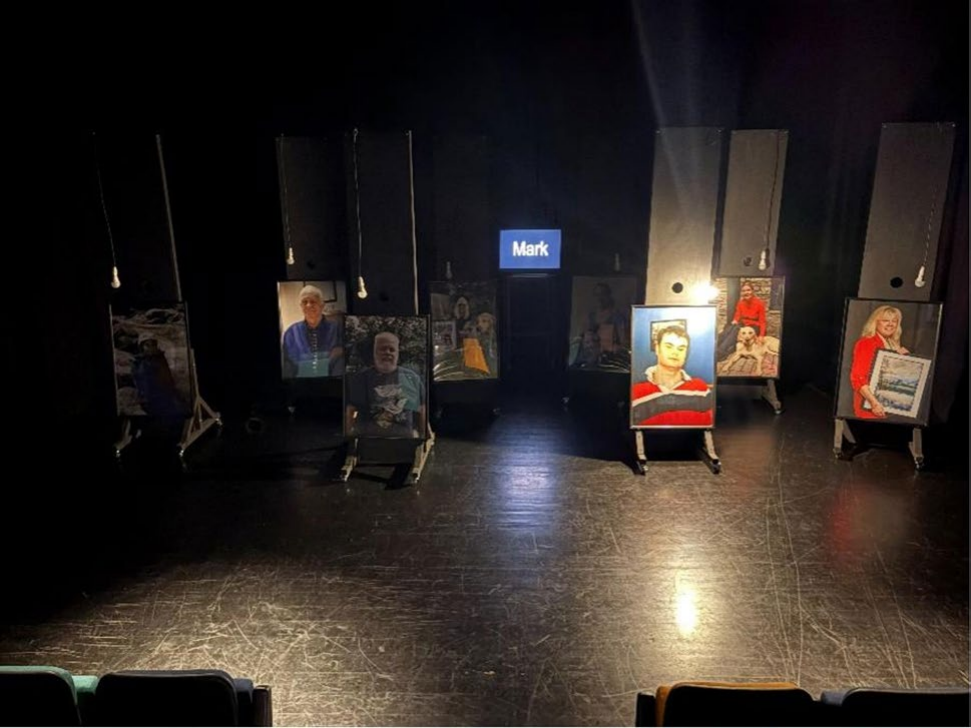
Eight voices in darkness installation at chapter, Cardiff

The installation took the form of eight personae (representing the eight project participants) occupying the stage within a darkened, black box theatre with raked seating (see Fig. 1.). The room was in low light to represent the literal darkness experienced by some people with sight loss and the metaphorical darkness of depression. Each persona was a two-and-a-half metre high, black, wooden panel with a loudspeaker fixed to the rear. Large framed photographic portraits of the eight participants leant against the structures at each base. A light bulb dangled in front of each portrait. The structures were placed loosely like characters on a stage. The voices of each participant could be heard separately in a random sequence from their respective loudspeakers. When they spoke their light came on and their forename was displayed on a monitor at the centre rear. A period of silence was chosen at random to place some reflection time between each speech segment. There was no overall fixed sequence.

The installation, *Eight Voices in Darkness*, was exhibited at an arts venue in Cardiff for 3 days (open to the public with free admission) to coincide with the Wales Annual Eye Care Conference and the Arts and Humanities Research Council’s (AHRC) Being Human festival.

Professionals attending the eye care conference were invited to view the installation and 20 people from vision-related professions also took part in an evening workshop to discuss their learnings. Attendees were split into groups and guided into discussions using reflective prompts adapted from Story Dialogue Method [19]. A similar workshop for mental health professionals not involved in the sight loss sector followed. Thirteen professionals, mainly from the National Health Service (NHS) and the mental health charity MIND attended.

Anonymous feedback was requested from all installation visitors and workshop attendees, with the options to submit handwritten feedback on paper slips, or online feedback via a QR link to JISC surveys. Two open-ended feedback prompts were provided - Having visited this installation: (1) What have you learnt? (2) What will you do differently in future?

To facilitate wider dissemination of the research, we created the first draft of a video with the portrait images and audio excerpts from the stories, along with some guidance for professionals on how to address mental health. This was played at the eye care professionals’ workshop to elicit feedback.

Ethical approval to collect and report feedback from visitors to the installation and workshop was received from Anglia Ruskin University (ETH2425-8820).

## Reflections & learning – from visitors and stakeholders

### Installation visitors feedback

The installation was attended by 186 visitors. Feedback was received from 92 visitors, and was coded inductively based on manifest content by KL (57 year old, sighted research optometrist) in NVivo (v14; Lumivero). Codes were then assigned to the Pendleton’s reflective questions of ‘what went well’, ‘what didn’t go well’ and ‘what have you learnt’.

#### What went well?

Visitors reported valuing the experience of the installation, with comments highlighting the value of hearing real life stories:

I appreciate these people sharing their stories to help the rest of us understand their experiences.

In the presentation of the installation, the darkness and silence between individual storytellers was found particularly powerful:

The black space were impactful and should have given people time to absorb and reflect.

Visitors noted the positive aspects of resilience and courage that they took from the installation. For example:

Blindness or vision impairment doesn’t mean that it’s the end of the world. It reminds us that there is still hope and these people are a living testimony to that. Their courage and persistence through anxiety and depression is inspiring.

#### What didn’t go well?

Some people found the installation a negative experience, commenting that they found it sad, miserable or too emotional:

It’s too emotional to sit through, we did for half hour.

A greater emphasis on positive messages was requested:

I do think it would be a good idea to also have more positive reflections about individuals getting on with their lives and doing amazing things as they have overcome issues in life.

#### What did visitors learn?

The key message reported by visitors to the installation was that they now had a better understanding of the impact of vision impairment on quality of life and mental health, thus demonstrating our research findings had been conveyed. For example:

This installation helps me understand the difficulties and mental health problems that patients with visual impairments are suffering from, which is not easy to imagine as a person with normal vision.

I hadn’t quite considered the emotional and mental health impact that having a visual impairment could cause. It really opened my eyes to the often unheard struggles people face

Beyond an improved general understanding of the impact of sight loss on mental health, visitors particularly noted the individuality of the experience of sight loss:

Different issues faced by everyone - impact of sight issues on mental health affects everyone differently.

Health professionals need to remember everyone is an individual.

The variety of experiences people have had and the different impact/s on their lives.

Visitors to the installation who were themselves visually impaired also noted appreciating individual experiences:

That my sight loss experiences are very similar to others, despite everyone’s journeys being very different.

About other people’s experiences with sight loss different from my own.

Visitors recognised the value of a range of support services to people with sight loss but also recognised barriers to getting assistance including awareness and availability of, and access to services. The impact of the installation in terms of what people reported they would do differently can be summarised as a desire to be more considerate. This included being more considerate as an individual:

Be more considerate and patient with others.

Not question why someone needs more time and space to get around

And in a work environment:

Consider ways of improving access to events for blind and partially sighted people.

Specific actions mentioned relating to becoming more considerate included intending to ask and / or listen more:

I want to be all ears and listen about the people with disability and how they carry on in life.

Not to be afraid to ask the question [about mental health].

And to be more empathetic:

To be more empathetic towards others and put myself in their shoes wherever necessary.

Some visitors with visual impairment reported being more likely to discuss their experiences and to support others in a similar position:

I now feel much more connected to the sight loss community which has been quite emotional. Thank you.

I will be more open to talking about my experiences and feelings. I will also look to be more accepting of my sight loss journey and work with charity sector & low vision practitioners to manage my progression more proactively and confidently.

I will remain positive and carry on doing my best to help and serve other in my peer group who need help and understanding.

Healthcare professionals who attended the first workshop additionally noted appreciation of the importance of interprofessional communication and referral:

Emphasise the importance of peer support, of timely information and the importance of all those in the sight loss/ophthalmology/optometry sectors understanding what everyone else in the sector does.

Mental health professionals who attended the second workshop, many of whom conduct telephone consultations, noted intention to consider sight loss within their practice:

Ask the people I see if they are visually impaired in first instance and how this impacts them and how I may or may not help with improving their experience.

### Storyteller (NC)

All storytellers were invited to reflect on being involved in the project. NC volunteered and was the only individual able to comment on sharing her story, visiting the installation and attending the workshop as she took part in all three. NC has been blind from birth and is a guide dog user.

#### What went well?

It was nice being able to tell people about the negative side of having a vision impairment, as highlighted in the research. Quite often when the public see individuals with vision impairment on the television it is in activities like the Paralympics and they see that they are courageous and resilient but don’t realise this comes at a price of working extra hard to get where they are. So, it was good to share the challenges too. I also found the organisation of the project went smoothly, from being introduced to the project at the workshop to taking part in the interview.

#### What didn’t go well?

I was disappointed that not many counsellors or psychotherapists turned up for the second workshop, the attendees were mainly mental health practitioners. As a psychotherapist myself, I would have liked to have seen more there, as they have a different role to mental health practitioners and need to understand the impact of sight loss on emotional wellbeing.

#### What did I learn?

It was nice to hear about others’ experiences and what it was like growing up for them with a vision impairment. It was also interesting to hear about losing sight, as I have been blind since birth.

### Researchers (CN, KL and RD)

#### What went well?

Involving people with lived experience through working with a charity (Sight Life) as a single point of recruitment was a positive experience. The charity was not only able to support the logistics of an introductory meeting, but it was also feasible to identify eight volunteers with different lived experiences. Further, as trust in and with the charity was already established, people were actively interested and willing to take part, and the storytellers were open in sharing their mental health challenges. Some storytellers reported enjoying the opportunity to share their story, and one reported positively that they had not previously had the opportunity to have someone sit and listen to them talking about the impact of their sight loss.

Involving the public voice in dissemination through art was powerful, with a lot of the visitors staying in the installation for a considerable time. The feedback demonstrated that the key things that people reported learning were broadly the key messages from our research.

Attendance at the installation was increased through tie-ins with the Wales Annual Eye Care Conference and the AHRC Being Human festival. This tie-in also allowed us to reach mental health professionals in the third sector who often have limited time or opportunity for taking part in professional development outside their immediate delivery needs, and who reflected positively on the opportunity to learn from the installation. We also reached an audience we had not specifically targeted in terms of people with sight loss, who did feed back that they benefited from the installation.

#### What didn’t go well?

A few visitors, storytellers and workshop participants were unhappy with different aspects of the installation. This was uncomfortable for the research team, who needed to manage their concerns and upset.

There was insufficient time within the project to go back to the storytellers with the edited versions of their stories before their inclusion in the installation. This led to upset for one storyteller who felt the most important part of their story had not been told, and one who was apprehensive about viewing the installation as she did not know what to expect.

Some healthcare professionals attending the workshop felt unhappy that storytellers reported negative experiences regarding interactions with services like those that they provided, when they tried hard to do the best job that they could with difficult circumstances, such as the delivery of bad news or of diagnoses of sight loss.

The first iteration of the video intended for wider dissemination was not well received by workshop participants. The group consensus was that we needed to portray a more balanced perspective, including the challenges of living with sight loss but also the solutions and positive experiences that professionals could learn from. Advice not to include narration or guidance from the researchers was given, instead the lived experiences should stand alone in conveying the key messages for health professionals. This highlights the importance of involving patients and professionals in creating research dissemination outputs. The feedback was extremely valuable in developing more appropriate content for wider dissemination and the video was revised into a final version and hosted alongside written resources.

#### What did we learn?

We learnt that peoples’ lived experience can be powerfully conveyed through art and can stimulate learning based on research findings. We were successful in conveying key messages heard across our research and in a personal way that resonated with the audience. Working with a charity facilitated identification of storytellers with diverse experiences and supported their involvement. Several individuals involved had enjoyed or benefited from taking part. Connecting with individuals with lived experience in this personal way has shaped the future direction of our research and two storytellers are already in involved in subsequent projects.

To enhance genuine and ethical involvement, in future we would allow more time and funding for storytellers to review and approve their edited contributions before finalising the installation [20]. It was beneficial to review the first draft of the video with the intended audience as this raised several issues which we could rectify before sharing widely.

Given the time commitment involved in creating the installation, incorporating a more permanent legacy in the form of the online video in addition to the shorter duration installation has been important to maximise the reach of the messages created.

Finally, we were reminded that involving individuals with lived experience in dissemination, and engaging a professional audience is an involved job which requires an additional set of skills to those required for everyday academic research. Researchers need training in these areas, and time to develop effective and ongoing partnerships, to realise the full potential of such projects.

### Artist (RB)

#### What went well?

There were several positive verbal responses about the installation’s form: (1) the space was relaxed and comfortable; (2) it had a church-like atmosphere; (3) there was time to absorb what was said.

Each narrator’s sequence was fixed chronologically but the random selection of each individual meant the combined sequence varied on each iteration. This allowed for the chance juxtaposition of themes within the discourse which is, to my mind, an important technique in installation art to enrich the flow of ideas. I was careful to avoid any modulation of the tone being presented, taking sizeable segments of speech without edits within those segments.

Feedback showed that the narratives raised awareness of the difficulties faced by vision impaired people, the variability of different needs and different eye conditions, and provided a view of sight loss that wasn’t the prevalent one found in the media.

#### What didn’t go well?

There was a frequently expressed negative response concerning the content of the narratives: it was too depressing/negative.

The content of the narratives was deliberately unresolved. I felt the presentation of the problems and challenges faced by the participants were the important data to convey to the visitor. This would avoid closing the conversation such that solutions are presented as an emotional and intellectual release valve within the installation. We wanted people to think about what they heard. This approach was determined largely by the material itself. The recordings were, on the whole, a picture of failures in services and prejudices that were hard to pass over.

#### What did I learn?

The work was an opportunity to engage with material that was beyond my control. The recordings and the photographs came to me in raw form, forcing a mode of presentation that took them at face value with little manipulation. Generally, I sculpt my materials into an aesthetic form that reaches a state of ‘exhibitability’. The products of that process are often taken to the next work for reuse and reworking. In the case of this piece, the organisation of the speech materials into chunks flanked by silence is something I would use again, as well as the quasi-theatrical arrangement of the personae.

## Conclusions

This project demonstrates the value of involving people with lived experience to communicate research findings in ways that are emotionally engaging, accessible, and meaningful for diverse audiences. The sound installation enabled storytellers’ voices to take centre stage and provided a powerful space for visitors to reflect on the mental health impact of sight loss. Feedback from public audiences and professionals showed that the installation deepened understanding, encouraged empathy, and prompted many to consider changes in their future practice.

The reflections across all the stakeholder groups highlight practical and ethical considerations in the feasibility of meaningful involvement of people with lived experience of sight loss in disseminating research findings through art. These include the need for adequate time for coproduction, accessibility and longevity of artistic formats, and careful support for both storytellers and audiences when dealing with sensitive content.

Overall, our learning reinforces that involving people with lived experience through arts-based approaches can enhance the reach and impact of research messaging on sight loss and mental health. However, doing so requires dedicated skills, resources, and relationships to be delivered responsibly. Continued collaboration with people with lived experience will be essential in developing future dissemination materials and training resources that further support practice change across vision and mental health services. The video is available as an educational resource on the Centre for Vision Services Research website [21].

## Data Availability

The datasets used and/or analysed during the current study are available from the corresponding author on reasonable request.
